# Dual Control for Jerk-Driven Robotics in Rehabilitative Planar Applications

**DOI:** 10.3390/mi11020141

**Published:** 2020-01-28

**Authors:** Francesco Aggogeri, Cinzia Amici, Nicola Pellegrini

**Affiliations:** Department of Mechanical and Industrial Engineering, University of Brescia, via Branze, 38, 25123 Brescia, Italy

**Keywords:** vibration, robotics, trajectory, jerk

## Abstract

This study compares a set of strategies to plan and control the trajectory of a robotic device in a planar workspace. These strategies are based on an affective application of jerk-laws able to indicate undesirable conditions (e.g., vibrations) facilitating the device control. The jerk is the time derivative of acceleration, and this solution provides an indirect means to control the variation rate of the actuator torques, while avoiding the complex robot dynamic models and their algorithms for computing the dynamics. In order to obtain a smooth trajectory, a regulator to control a robotic device has been developed and validated. It consists of the implementation of two control modules able to (i) generate the predefined trajectory and (ii) guarantee the path tracking, reducing unwanted effects. In this case a simple S-shaped path has been originated by the “trajectory generator module” as a reference movement to rehabilitate upper limb functionality. The numerical simulation and the results of preliminary tests show the efficacy of the proposed approach through the vibration smoothness appraisal associated with the motion profile.

## 1. Introduction

The application of robotics in progressing assistive and rehabilitative movement function recovery has gained significant momentum in recent years. Clinical demonstrations, in restoring functions for upper extremity movements, are now requested in stroke and aged populations. The progress of the existing robotics system to recover lower- and upper-limb functionalities represents a promising technique to realize accurate recovery training, allowing high frequency-exposure and repetitions while reducing medical personnel cost by enabling the prospect to assign one therapist to train two or more patients [1]. Moreover, robotics offers consistent advantages in combining low cost solutions with the ability to execute different exercises realizing a task-driven therapy on the same device. 

These results may be reached through the assisted-as-needed (AAN) control strategy. In this concept, the aim of the robotic device is to either assist or correct the movement of the user [2,3]. Examples of the AAN approach are collaborative robots (cobots) [4], which are able to manipulate items in cooperation with a human subject. In collaboration mode, the required therapies are usually performed by manipulating items in cooperation between the robot and patient [3,4]. The human subject is in physical contact and interacts directly with the robot, exchanging mutual data. The patient applies forces following the preferred path and the robot supports the user through its gestures along the workspace. In this way, the movement generated by the human’s intelligence is shared with the machine’s mechanical power in executing the manipulation task, as the robot provides the necessary power to complete the required task. 

The robot collaboration has to guarantee the user’s safety during the physical activity, avoiding the intermittent movement of the patient. This level of safety may be increased by the control standpoint, ensuring that the automation avoids any improper action or upsetting effects due to resistive forces of the robot [5]. In addition, when the therapist or patient pushes the end-effector, the robot has to guarantee that the end-effector follows the correct movement. The main risk in the robotic application is to incur trajectory errors, compromising the rehabilitation training and patient’s safety. The robotic device (e.g., end-effector) needs to be effectively controlled, ensuring both a smooth trajectory and vibration containment due to patient hesitations.

This study proposes a set of strategies to control the therapy trajectory of a Cartesian mechanism in a planar workspace. These strategies are based on an affective application of jerk-laws. 

The proposed regulator is able to control the robotic device obtaining a smooth trajectory. It consists of the implementation of a set of modules to generate an appropriate rehabilitation path and guarantee the tracking accuracy of the generated trajectory. The controller uses a feedback loop configuration, measuring and regulating the forces and accelerations exerted by the user on the end-effector. Additionally, the regulator limits the kinetic energy power provided by the robot movement and imposes the speed profile limit to guarantee the user’s safety. In this way, the proposed solution avoids stiff application of mechanical components, which are usually adopted to contain the vibrations that influence the dynamical performances due to the increase of the system mass [6]. The regulator has been developed for the XY plane but it may be extendible to other dimensions.

The novelties of the contribution are: (i) the application of well-known industrial robotic concepts in rehabilitative application; (ii) the dual-control development is based on the trajectory generator module that imposes the requested path (S-shape, cycloidal, trapezoidal, etc); (iii) user-safety is guaranteed from a performance index for misusage, in fact, the jerk-based index is also hesitation-sensitive for non-expert subjects; (iv) the solution provides the indirect means to control the variation rate of the actuator torques, avoiding the complex robot dynamic models and their algorithms for computing the dynamics; and (v) the invariance from dominating modal frequencies could be an opportunity to propose a flexible, light, and wearable device that patients could use at home.

The paper is organized as follows: Section 2 gives a brief description of rehabilitation requirement and discussion of S-curve following. Next, the problem formulation related to the robot manipulator and the selected jerk S-curve trajectories are described in Section 3. The strategy for controlling all movements of mechanisms is presented in Section 4. In Section 5, numerical simulations and experimental results on rehabilitation manipulator are addressed. Finally, our conclusions are drawn in Section 6.

## 2. Rehabilitation Requirements and Research Opportunities

**Figure 1:**
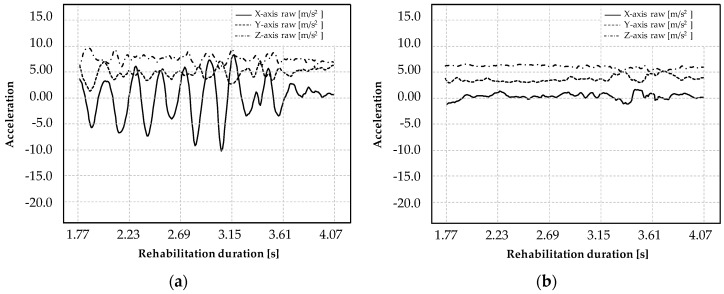
Simulation of the S-shape trajectory executed by a mimicked disease user (**a**) and a healthy user (**b**).

In the rehabilitation therapy, an S-shape trajectory task is used as a reference movement to rehabilitate upper limb functionality. Usually, the required motion time takes 2.0 s from the start point to the final point in the XY-plane. Figure 1a shows the simulated performance of a diseased subject, highlighting a significant number of perturbations in the X-axis direction that may compromise the trajectory. These conditions are typical of patients affected by hesitations and tremors with a frequency range from 4 to 6 Hz, such as Alzheimer’s and Parkinson’s diseases or stroke survivors. Figure 1b illustrates the same task executed by a healthy subject. Figure 1b displays a smooth trajectory with a limited effect of perturbations in comparison to the mimicked movement recorded in Figure 1a, in particular, along the X-axis direction. The motion laws should satisfy the therapist’s requirements, in accordance to the power of the actuator, avoiding any vibration and resonance frequency mode. The aim is to support the user, avoiding deviations from the required path, controlling the movement and the velocity through the robotic device.

## 3. Problem Statement: The Robot Manipulator and the Analyzed Jerk Profiles

**Figure 2:**
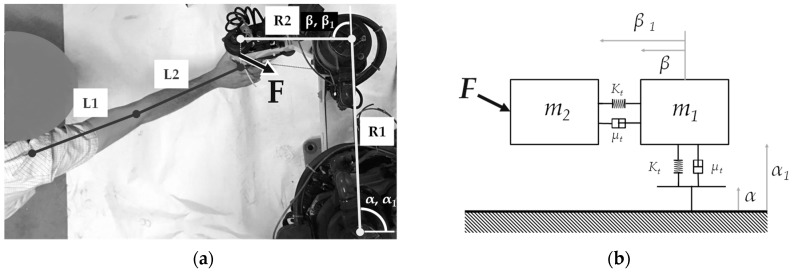
Device architecture for the rehabilitation of planar movement (**a**), generic lumped dynamics model (**b**).

The proposed manipulator aims to reproduce a rehabilitation training of the upper limbs. It has to satisfy the therapist’s requirements, performing the errorless training, as shown in Figure 2. 

The working principles of the robot-assisted therapy consist of:

- supporting the patient limb payload for the duration of the rehabilitation exercise;
- compensating for the errors when deviations or hesitation-perturbations of movement are measured.

The trajectory deviations and the motion perturbations are the main causes of an unsuccessful rehabilitation training. Using the kinematic model developed in previous works [7,8], the rehabilitation training may be performed by a SCARA robot. The Cartesian reference O(x, y) defines the limb position, identified by angles (α, β) where R1, R2 represent the link distances between the robot-patient interface and the reference origin, respectively. Figure 2a shows the system configuration and an example of the resultant vector of the end-effector movement. In order to obtain the proper external forces by joint, an inverse dynamics study has been developed on the mechanical system, as stated in Figure 2b.

According to the notations of Figure 2, the movement equations are described by:

(1) $$\frac{V_{m2}(s)}{F(s)}=\frac{1+\frac{2\cdot \Psi_{n}}{\omega_{n}}s+\frac{1}{\omega^{2}{}_{n}}s^{2}}{m_{\mathrm{tot}}\cdot s\cdot \left(1+\frac{2\cdot \Psi_{n}}{\omega_{n}}s+\frac{1}{\omega^{2}{}_{n}}s^{2}\right)};$$

(2) $$\frac{V_{m1}(s)}{V_{m2}(s)}=\frac{1+\frac{2\cdot \Psi_{n}}{\omega_{n}}s}{1+\frac{2\cdot \Psi_{n}}{\omega_{n}}s+\frac{1}{\omega^{2}{}_{n}}s^{2}}.$$

The equations describe the transfer functions between the force exerted by the robot on the user and the velocity vector of the mass-ith. The static friction is not considered in the present work. The parameters are defined as follows:

- *F*: the force exerted by the robot on the user through the end-effector,
- *m*_2_: the mass of the SCARA secondary arm,
- *m*_1_: the mass of the SCARA primary arm,
- *m*_tot_: the mass of system,
- *V*_mi_: the velocity vector of the i-th mass,
- *ω_n_*: the natural pulsation, and
- Ψ: the damping ratio (Ψ).

Considering a cascade control, the position transfer function between the estimated reference movement (*x*_ref_) and the actual motion (*x_m_*_1_) takes the following form:

(3) $$\frac{x_{m1}(s)}{x_{\mathrm{ref}}(s)}=\frac{1+\frac{G_{\mathrm{FW}}}{G_{x}}s}{1+\frac{1}{G_{x}}s}\frac{1+\frac{2\cdot \Psi_{n}}{\omega_{n}}s}{1+\frac{2\cdot \Psi_{n}}{\omega_{n}}s+\frac{1}{\omega^{2}{}_{n}}s^{2}};$$

where *G_x_* and *G*_FW_ define the position and feedforward coefficients, respectively.

The potential discontinuity of the end-effector movement may generate the resonance modes of the device. The transitory error, *ε* (*t*), of the motion is defined as follows:

(4) $$\varepsilon (t)\equiv x_{\mathrm{ref}}(t)-x_{m1}(t)$$

This error is characterized by two components: aperiodical, *ε*_ap_(*t*), and periodical, *ε*_vib_(*t*), terms. The aperiodical term is generated by the incorrect identification of the regulator parameters, while the periodical contribution is produced by the vibrations of the rehabilitation device. The intrinsic damping of the modal modes is not considered in this work. In previous studies [8,9,10,11], it has been demonstrated that the system has the maximum hesitation when the acceleration of the device is constant. For this reason, a set of trigonometric function has been applied to the jerk profile in order to evaluate the most suitable law to reduce the deviation from the trajectory tracking. 

**Figure 3:**
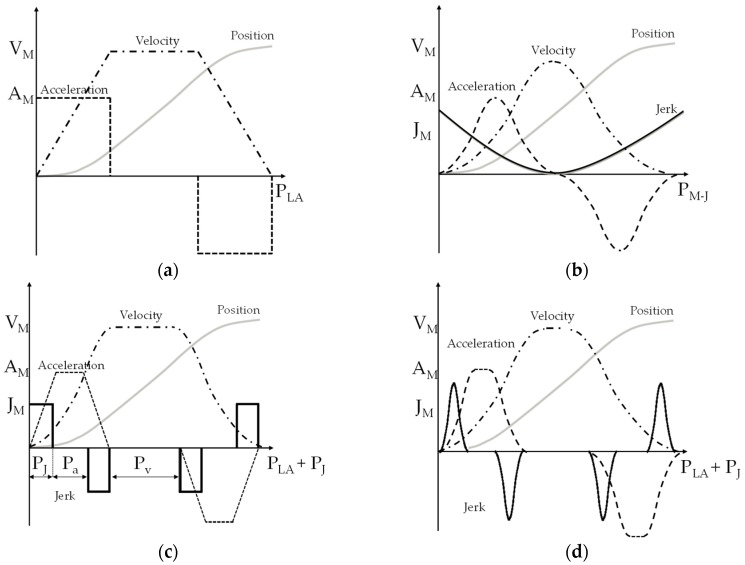
Jerk S-curve laws: constant acceleration (**a**), minimum jerk (**b**), limited jerk (**c**), harmonic jerk (**d**) [12].

A number of jerk-laws are stated in Figure 3: constant acceleration (a), minimum jerk (b), limited jerk (c), and harmonic jerk (d). The graphical representation shows the relation between V_M_, A_M_, and J_M_ (Velocity, Acceleration, and Jerk) with the generic period measured in time-domain P_V_, P_A_, and P_J_ (Period of time with constant-velocity, Period of time with constant-acceleration, and Period of time with constant-jerk). The minimum-jerk motion duration is represented in Figure 3a–d by P_M-J_, while the execution time based on limited acceleration is noted as P_LA_. 

The study of the jerk profile permits us to minimize the effect of the intrinsic modes. When comparing the jerk models shown in Figure 3, the minimum-jerk is the profile that minimizes the area, *J*(*x*_ref_(*t*)), under the jerk-law, obtaining the smoothest velocity profile. *J*(*x*_ref_(*t*)) is considered as a performance index, and is formulated as follows:

(5) $$J\left(x_{\mathrm{ref}}(t)\right)=\frac{1}{2}\cdot \int_{0}^{P}{\dddot{x}}_{\mathrm{ref}}^{2}(t);$$

where *P* is the specified execution time. The min-jerk movement is the most applied model in slow motion applications [12,13,14] as the resultant trajectory well represents the human motion. 

## 4. Dual Control Strategy Development

**Figure 4:**
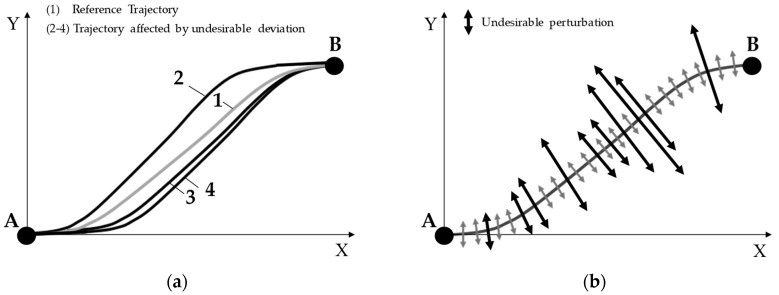
The control strategy aims: trajectory generation (**a**) and trajectory tracking (**b**).

The control of the end-effector is based on the simultaneous application of two functions [15,16,17,18,19], as shown in Figure 4. The first function aims to generate a correct S-shape trajectory from the starting point A to the final point B (Figure 4a), while the second function guarantees the accuracy in trajectory tracking, reducing any potential perturbation (Figure 4b).

**Figure 5:**
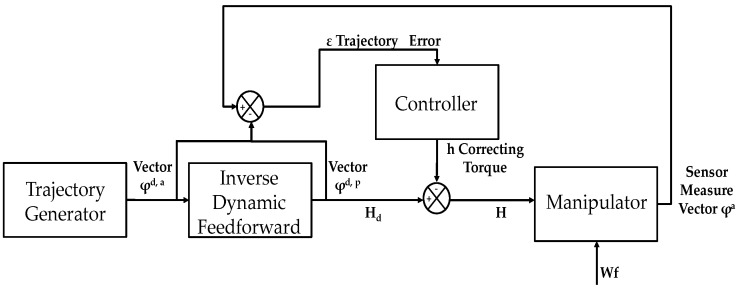
The main control architecture of the system.

Figure 5 shows the main configuration of the control system. It consists of a feedback control supported by an inverse dynamic feedforward model. Combined feedforward and feedback control can significantly improve tracking accuracy performance. In this case, a sensor, located on the end-effector, measures a signal (e.g., acceleration, velocity), represented by vector φ^a^. Comparing the feedback signal with the generated reference trajectory (vector φ^d,a^), the ε error is estimated for the compensation. Furthermore, a feedforward control permits the model to suppress the disturbances before they influence the required trajectory. The control modules are organized in accordance to the structure, as illustrated in Figure 5, where φ^a^ is the vector of the actuated-degrees-of-freedom (DOFs), φ^p^ is the passive-DOF vector, while H defines the control matrix.

In particular, the “trajectory controller generator” converts the force exerted by the user in the displacement directions for all actuated DOFs. A force threshold may be regulated in order to transfer the suitable force to the user for rehabilitation training. In this way, the system may also support the user when the resultant capacity is not sufficient to reach the target point or to reach the correct direction when the reference trajectory is not satisfied. Finally, the actuators are regulated by a torque-based control, which manages the required forces to satisfy the trajectory of the active DOFs [20,21,22,23,24,25]. The aim is to determine the nominal torque *H_d_* and the reference path for ϕ^p^, denoted ϕ^d,p^, so that ϕ^d,T^ satisfies the motion equations in (6). In addition, a force adjustment, *h*, is added to the model to ensure the tracking requirement, and this corresponds to the output of the required regulator, as follows:

(6) $$H=h+H_{d}$$

## 5. Simulation and Preliminary Experimental Results

**Figure 6:**
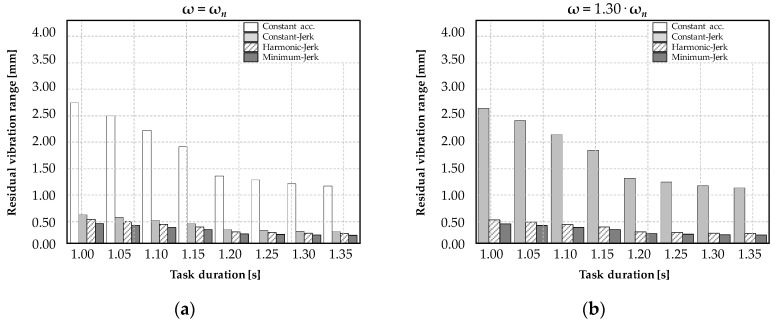
The time required for vibration reduction of the jerk-controlled laws with *ω* = *ω_n_* (**a**) and *ω* = 1.30·*ω_n_* (**b**).

In order to validate the controller, a set of simulations has been executed considering the proposed jerk-laws [8]. The jerk value has been estimated and evaluated by joint (DOF), imposing the reference trajectory (S-shape). Considering that the time to suppress the oscillations is influenced by the natural period of the dominating mode [12], Figure 6 compares the time required to reduce vibrations of the Cartesian robot with *ω* = *ω_n_* and ω = 1.30∙*ω_n_*, by the jerk profile.

Figure 6 demonstrates that the control of the jerk is preferred to acceleration in vibration reduction (magnitude and time). The results also underline that the control strategies are independent from the robotic kinematics and features. In particular, the proposed comparison shows that Minimum-Jerk is the most suitable control method to contain vibrations when the robot device operates closing to the modal frequency. These results encourage the use of jerk in the rehabilitation device control, as unpredictable conditions or incorrect estimation of the dominating frequency of the co-manipulator system may occur. In fact, the minimum jerk method is able to contain hesitations effectively, when vibrations (e.g., patient tremors) have a frequency range close to the dominant modal frequency (e.g., *ω* = 1.30∙*ω_n_*). Table 1 summarizes the obtained results of simulations using the jerk-laws. The control strategy of the minimum and harmonic jerk is able to obtain a residual vibration amplitude lower than the 0.24 mm range in 0.35 s. These results are promising to guarantee a smooth and accurate trajectory from the user during the rehabilitation exercises.

**Figure 7:**
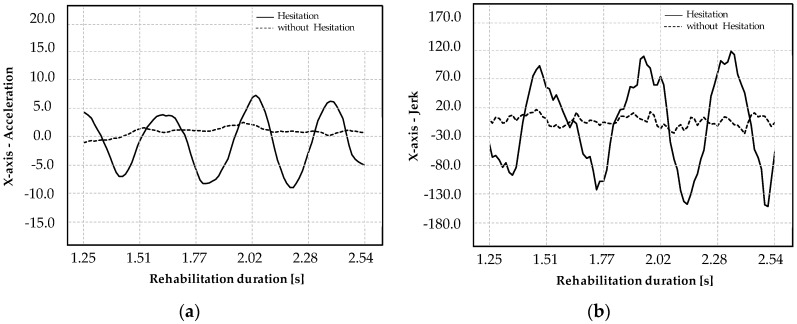
Rehabilitation S-shape exercise with jerk-based measures: accelerations measured and controlled on the end-effector (**a**); jerk observed and controlled on the end-effector (**b**).

A set of experimental tests was performed to validate the proposed regulator applied to a Cartesian robot. The tests consisted of the execution of a point-to-point S-shape trajectory, assigning the start and the target points. Figure 7a compares the accelerations measured on the end-effector during a set of tests executed by a healthy subject (male, 24 years old) and a user (mimic) simulating a diseased subject performance. In Figure 7b, the profile of the acceleration is evaluated comparing the tests executed by a disease mimicking-user considering on–off control. The tremor imitation is denoted by the solid line while the dotted line describes the acceleration resultant when the regulator is activated. These results show the effective compensation of the vibrations thorough the control of the jerk that is more sensitive to tremors and hesitations. 

## 6. Conclusions

This paper presents a dual-controller implemented in a traditional robotic device to assist the patient in functional errorless learning in a planar workspace. The proposed regulator has been designed and tested to guarantee rehabilitation path tracking to achieve both hesitation supervision and reduction. A comparison of a set of strategies to control the trajectory of the robotic device is described. These strategies are based on the application of jerk-laws, that is, a parameter, more sensible than others (e.g., speed or acceleration), able to reduce undesirable conditions (e.g., a patient’s sub-movements, or hesitations) facilitating the device control. Numerical results confirm that the most promising residual vibration strategy is a minimum-jerk-law. Simulations also show the efficacy in unpredictable conditions, when it is present as a bias in the estimation of the dominating frequency of a co-manipulator system. 

The current approach was limited to the case of planar Cartesian mechanisms, with motions that are not combined and can be accomplished independently. Preliminary trials realized on lab-equipment show the approach efficacy through the observations of the mimic tremor scenario. It is possible to confirm the role of proposed jerk-based indices, as an important predictor of functional motion restoration of the limb. Moreover, the invariance from dominating modal frequencies could represent an opportunity to propose a flexible, light, and wearable device that a patient could use at home. Future activities will be developed to verify the efficacy in multi-trajectory tracking and extending the research with a tridimensional workspace involving obstacle avoidance.

## Figures and Tables

**Table 1:** The residual vibration range (mm) of the jerk-laws of a Cartesian robot.

Movement Laws	Residual Vibration *ω* = *ω_n_*	Res. Vibration Freq. Bias 30% *ω* = 1.30·*ω_n_*
Limited acceleration	2.51	-
Limited Jerk	0.44	2.42
Harmonic Jerk	0.42	0.37
Minimum Jerk	0.38	0.24
